# Copper metabolism and cuproptosis: broad perspectives in the treatment of hepatocellular carcinoma

**DOI:** 10.3389/fonc.2025.1555858

**Published:** 2025-07-30

**Authors:** Jiale Liang, Ruting Wang, Hongxi Wu, Zhenjin Huang, Ruohan Zhang, Feng Jiang

**Affiliations:** ^1^ Graduate School, Guangxi University of Chinese Medicine, Nanning, Guangxi, China; ^2^ Ruikang Hospital, Guangxi University of Chinese Medicine, Nanning, Guangxi, China

**Keywords:** hepatocellular carcinoma, copper, cuproptosis, cuproptosis-related genes, copper chelators, copper ionophores

## Abstract

Copper is an essential trace element that plays a pivotal role in multiple biological processes, including energy production and angiogenesis. It is also a vital cofactor necessary for the maintenance of biological functions and has been implicated in cancer development. The recently identified form of cell death, cuproptosis, has a unique induction mechanism—accumulated copper ions directly bind to lipoylated proteins in the mitochondrial tricarboxylic acid (TCA) cycle, triggering toxic protein aggregation and cell death. This process can be specifically induced by oxidative stress and mitochondrial dysfunction, providing a novel direction for the development of anti-tumor strategies that target copper metabolism. In hepatocellular carcinoma (HCC), there is a significant correlation between disturbances in copper metabolism and abnormalities in the cuproptosis pathway. HCC cells maintain pro-carcinogenic copper levels through the upregulation of copper transporter proteins such as copper transporter 1 (CTR1). Conversely, the dysregulation of the expression of key genes involved in cuproptosis (ferredoxin 1, lipoic acid synthetase) may mediate treatment resistance. In this review, we focus on the mechanism by which cuproptosis influences the occurrence and development of HCC, evaluate its potential as a diagnostic biomarker, and examine therapeutic strategies targeting this form of cell death (nanocarrier-based delivery of copper ion carriers, CRISPR-mediated editing of copper-regulated genes). These strategies may provide a novel perspective for overcoming the current therapeutic limitations of HCC.

## 1 Introduction

Liver cancer ranks among the six most prevalent malignant neoplasms worldwide (1). Histologically, liver cancer is classified into focal nodular hyperplasia (FNH), cholangiocellular carcinoma (CC), hepatocellular adenoma (HCA), hepatocellular carcinoma (HCC), and combined hepatocellular carcinoma (2). Among these, HCC is the predominant type, accounting for approximately 85% of all cases of liver cancer (3). At present, surgical removal and liver transplantation are the preferred treatment approaches for HCC. However, the lack of clear symptoms during the initial stages of HCC, coupled with the inadequate specificity and sensitivity of relevant diagnostic tests, results in most patients being diagnosed with advanced disease. This renders surgical and local treatment options challenging to implement (4, 5). Moreover, owing to primary and acquired resistance, most patients with advanced HCC derive little long-term benefit from systemic therapy. Overall, the outcomes of HCC treatment remain largely unsatisfactory (4).

Copper is an essential metal nutrient for all living organisms. It participates in a variety of biological processes, playing a crucial role in metabolic and physiological functions such as metabolism, nerve stimulation, blood cell formation, and the production of connective tissue, among others (6). Recently, Tsvetkov et al. identified a novel type of cell death, termed cuproptosis, whose mechanisms are distinct from those of other known forms of cell death, such as apoptosis, necroptosis, and ferroptosis. Notably, in cuproptosis, excess copper accumulation triggers proteotoxic stress, leading to cell death (7). The liver plays a key role in copper uptake, storage, and excretion, making it susceptible to imbalances in copper levels, which can lead to the development and progression of liver-related diseases (8). This property of the liver makes cuproptosis a promising target for the treatment of such diseases, especially HCC. Here, we summarize the relationship between copper metabolism, cuproptosis, and HCC, aiming to guide future cancer treatment.

In this review, we systematically examined studies investigating the role of cuproptosis in HCC, including its underlying mechanisms, as well as therapeutic strategies that target this form of cell death in HCC treatment. We undertook a literature search using standardized PubMed/Web of Science terminology under the Medical Subject Headings (MeSH) term “Carcinoma, Hepatocellular” along with 19 variants in the title/abstract field (e.g., “Hepatocellular Carcinoma” and “Hepatoma”). The copper-related terms included the MeSH subject term “Copper” [MeSH], the free-text terms “Copper” and “Copper-63”, and “cuproptosis” (#4–#7). The two sets of concepts were linked *via* the logical operator “AND” (#8) (the full strategy is provided in **Supplementary Table S1**). The main inclusion criteria were original studies and reviews that explored the mechanisms or prognostic relevance of copper metabolism and cuproptosis in HCC. To ensure relevance, we prioritized literature published in the last five years (2020–2025), while also incorporating relevant older seminal works.

## 2 Copper metabolism

### 2.1 Physiological copper metabolism

**Figure 1:**
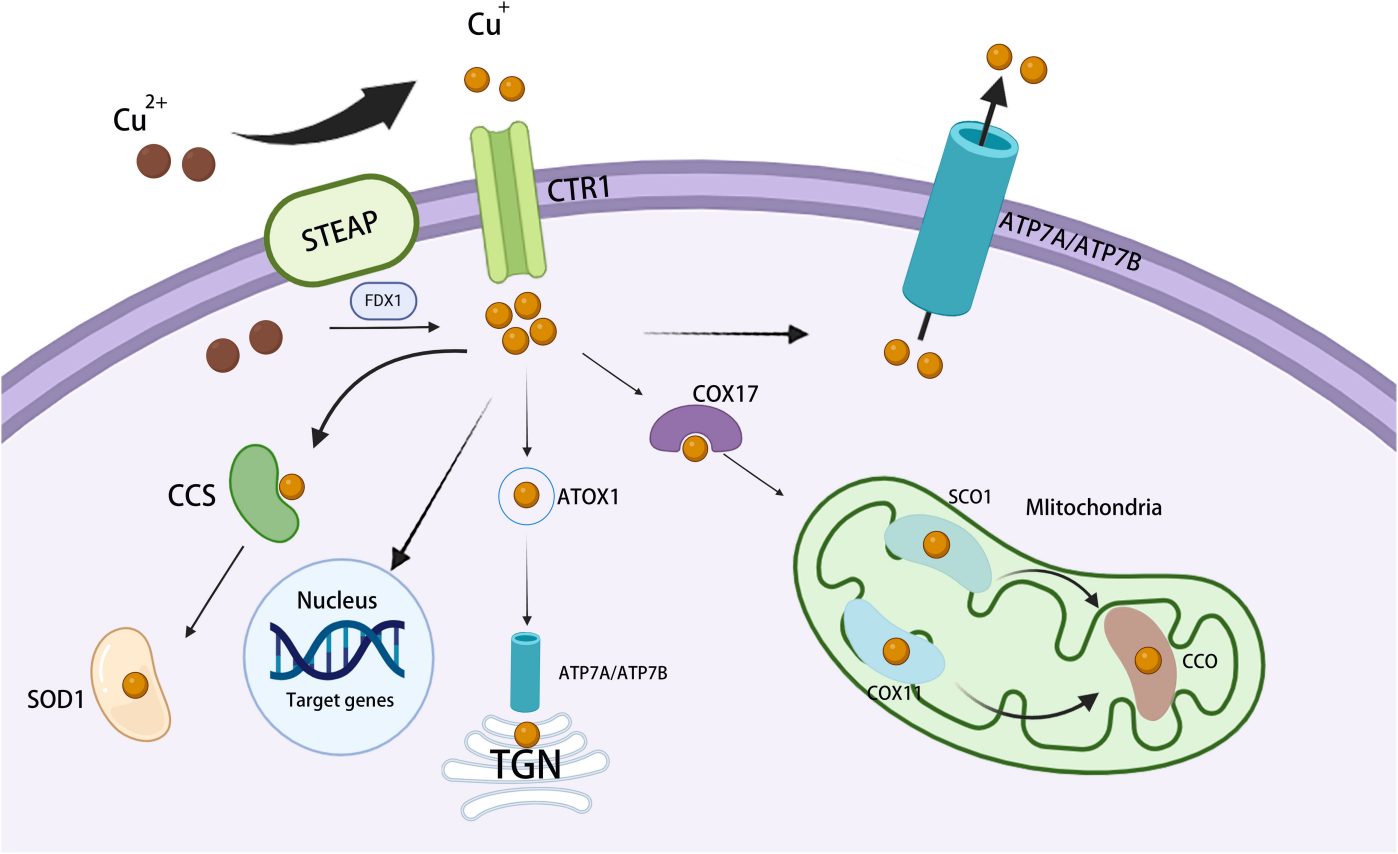
Overview of cellular copper metabolism. Extracellular Cu^2+^ is converted to Cu^+^ by STEAP and is then transported across the cell membrane by CTR1. Inside the cell, cytosolic Cu chaperones, such as CCS and SOD1, bind Cu^+^ and assist in transporting it to distinct subcellular compartments. In mitochondria, Cu^+^ connects with CcO, thereby participating in the respiratory chain and redox processes. In the mitochondrial intermembrane, COX17 binds Cu^+^ and transfers it to SCO1 or COX11, aiding the inclusion of Cu^+^ into cytochrome oxidase subunits. In the TGN, ATP7A and ATP7B transfer Cu^+^ from the cytosol to the TGN lumen, thereby activating Cu-dependent enzymes in the secretory pathway. In the nucleus, Cu^+^ binds to transcription factors and drives gene expression. STEAP, six-transmembrane epithelial antigen of the prostate; ATP7A/B, copper-transporting ATPase 7A/7B; CcO, cytochrome c oxidase; CCS, copper chaperone for superoxide dismutase; COX11, cytochrome c oxidase 11; CTR1, copper transporter 1; SCO1, synthesis of cytochrome c oxidase 1; SOD1, superoxide dismutase 1; TGN, *trans*-Golgi network.

Copper is a vital trace element for humans and is involved in a wide range of biological activities. Living organisms carefully regulate copper metabolism to maintain copper levels within a healthy range (9) (**Figure 1**).

Dietary intake constitutes the primary copper source, and copper is usually absorbed *via* the intestinal tract, primarily the duodenum (10). Within the mammalian digestive system, copper predominantly exists in the Cu^2+^ state. This form of copper can cross the intestinal barrier *via* divalent metal transporter protein 1 (DMT1), but is not directly available for use in metabolic processes (11). To become biologically active, Cu^2+^ must be reduced to Cu^+^, a process that is mediated by prostate six-segment transmembrane epithelial antigen (STEAP) and duodenal cytochrome b (DCYTB) (12–14). Cu^+^ enters the gastrointestinal tract and binds to the copper molecular chaperone antioxidant 1 (ATOX1), which facilitates its transport across the epithelium (15, 16).

ATP7A and ATP7B are key regulators of copper levels and play both distinct and complementary roles in hepatic copper metabolism. ATP7A exports copper from the intestinal epithelium into the bloodstream, allowing its efficient distribution to various tissues and organs. ATP7B, expressed mainly in the liver, excretes excess copper into the bile, and this copper is subsequently removed from the body (17). Elevated ATP7A and ATP7B levels in HCC are associated with resistance to cisplatin and carboplatin, as well as poor patient prognosis (18). In hepatocytes, the copper-binding proteins metallothionein 1 (MT-1) and MT-2 play a pivotal role in copper storage in the liver (19). Notably, although MT-3 shares a similar metal-thiolate cluster with MT-1/2 and is also capable of modulating copper, its primary site of action is in neuronal cells rather than the liver (20). Altered expression of MT-1 and MT-2 may contribute to HCC development and progression, and may serve as prognostic markers in HCC patients (21).

Once in the cytoplasm, copper transportation is orchestrated by a network of high-affinity copper chaperones, which facilitate its movement to various destinations within the cell, including the Golgi apparatus, mitochondria, and the nucleus (22). Copper chaperone for superoxide dismutase (CCS) facilitates the transport of copper to specific proteins that regulate the distribution of SOD1 between the cytoplasm and the inner mitochondrial membrane (IMM) in an oxygen-dependent manner. This mechanism helps maintain reactive oxygen species (ROS) homeostasis (23). The copper metallochaperone ATOX1 translocates copper to the *trans*-Golgi network (TGN) (22). ATOX1 binds copper and delivers it to the copper-dependent ATPases ATP7A and ATP7B located in the Golgi network, thereby promoting the synthesis of copper-containing enzymes such as lysyl oxidase (LOX) and cuprocyanin (CP), and maintaining dynamic copper homeostasis (24). The helper protein Cox17 facilitates the transfer of copper to Sco1, Sco2, and Cox11, with Sco1 and Sco2 being pivotal in the assembly of cytochrome c oxidase (25). In mitochondria, copper supports oxidative phosphorylation (OXPHOS) and mitochondrial function through its interaction with cytochrome c oxidase (COX) (22). Current studies suggest that key copper chaperone proteins greatly contribute to malignant processes such as tumor proliferation, metastasis, and resistance to cuproptosis through the sustained delivery of copper to cancer cells (26, 27). Targeting the ATP7A/ATP7B/MT-1/2 pathway may represent a therapeutic strategy for copper-related malignancies.

A variety of experimental models relating to copper metabolism have been established, including a TX mouse model of Wilson’s disease (WD) and a HCC xenograft model. These models have revealed the significant effect of copper dishomeostasis on the development of liver diseases, especially HCC, and provided a theoretical basis for interventions targeting copper metabolism to address the disease at both the systemic and cellular levels.

### 2.2 Copper anomaly

Copper metalloenzymes and related molecules with roles in copper metabolism participate in numerous essential cellular processes, including antioxidative reactions, iron transport, neural signal transmission, epigenetic modification, and the crosslinking of elastin and collagen. Dysregulation of copper metabolism can have detrimental effects on cells (28).

WD and Menkes’ disease (MD) are typically associated with abnormally elevated or decreased copper levels resulting from ATP7A- or ATP7B-related dysfunction, respectively. WD is caused by decreased copper excretion due to abnormal copper metabolism and ATP7B dysfunction. This also puts patients with WD at increased risk of HCC due to copper accumulation in the liver (29). MD is a condition caused by a lack of copper due to mutations in the *ATP7A* gene (30). The absence of functional ATP7A results in the gut’s inability to absorb copper, consequently leading to significant overall copper deficiency (9). As 95% of copper in the body is excreted through the liver, excess copper accumulation in this organ can cause varying degrees of liver damage and potentially also HCC development (31). Recent studies suggest that copper overload may promote hepatocarcinogenesis through mechanisms such as oxidative stress, mitochondrial dysfunction, and aberrant activation of signaling cascades such as the MAPK and PI3K/AKT signaling pathways (32, 33). However, these mechanisms require further validation in clinical settings.

## 3 Molecular mechanisms underlying the effects of copper on cuproptosis and other forms of cell death

### 3.1 Copper and cuproptosis

**Figure 2:**
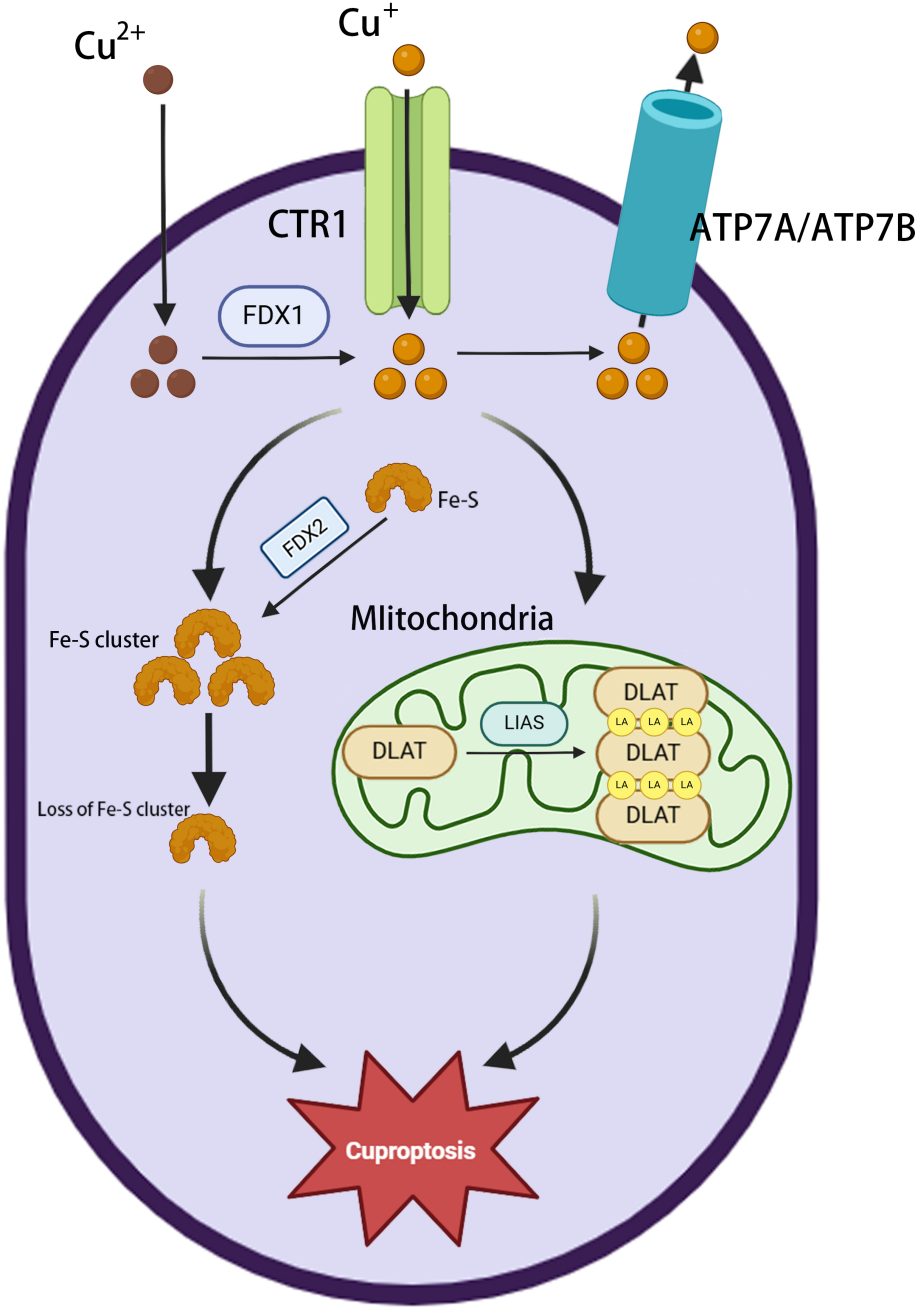
Overview of cuproptosis. Extracellular Cu^2+^ enters the cytoplasm *via* the copper transporter protein CTR1 and is then reduced to Cu^+^ by FDX1. ATP7A/ATP7B mediate copper efflux to maintain homeostasis. Cu^+^ accumulation in mitochondria triggers the loss of Fe-S clusters (FDX2 is involved in regulation); LIAS is inactivated, blocking lipid acylation metabolism; and Cu^+^ binds to DLAT, inducing its abnormal oligomerization and aggregation, leading to cuproptosis.

The major morphological features of cuproptosis include plasma membrane rupture, mitochondrial shrinkage, and endoplasmic reticulum damage (28). The underlying mechanism involves the abnormal accumulation of copper in the mitochondria of affected cells. In excess, the binding of copper to lipoylated proteins induces their aggregation, causing proteotoxic stress and ultimately cell death (34). Ferredoxin 1 (FDX1) is a key factor in cuproptosis (35), providing electrons to the enzyme lipoic acid synthase (LIAS), thereby initiating a free radical chain reaction necessary for the LIAS-mediated biosynthesis of lipoyl cofactors (36). Cells deprived of FDX1 and LIAS can resist copper-dependent cell death, thus highlighting the correlation between FDX1, protein lipid acylation, and copper toxicity. FDX1 collaborates with LIAS to facilitate the formation of a disulfide bond in dihydrolipoamide S-acetyltransferase (DLAT), which is a key step in triggering its abnormal oligomerization (37). This disrupts the pyruvate dehydrogenase complex (PDC), resulting in the impairment of the TCA cycle and, in turn, ROS generation. Furthermore, copper has been shown to have a significant disruptive effect on the mitochondrial [4Fe-4S] cluster assembly pathway, reducing the production of Fe-S cluster proteins by competing with Fe/Fe-S clusters for the same protein metal binding sites (38). During mitochondrial respiration, Cu^+^ can also directly bind to lipoylated components of the TCA cycle (37), leading to the accumulation of lipid-acylated proteins and a reduction in the production of iron-sulfur cluster proteins, resulting in proteotoxic stress and, ultimately, cell death (37). The phenomenon of cuproptosis provides compelling evidence for a direct correlation between copper toxicity and mitochondrial function (**Figure 2**).

Mitochondrial respiration plays a pivotal role in promoting tumor progression by supplying substantial levels of energy to tumor cells and thereby preserving their stemness (39). Li Z et al. discovered that MELK promotes DLAT expression *via* the PI3K/mTOR pathway. This regulatory effect contributes to improved mitochondrial respiration, the elimination of excessive intracellular ROS, and the reduction of intracellular oxidative stress/injury. Ultimately, these effects contribute to the development of HCC (40). AT-rich interactive domain-containing protein 1A (*ARID1A*), encoding a subunit of the switch/sucrose non-fermentable chromatin remodeling complex, is one of the most commonly mutated genes in HCC (41, 42). In ARID1A-deficient HCC cells, glycolytic activity is markedly reduced, and mitochondrial respiration is increased. Additionally, the loss of the expression of genes related to the TCA cycle makes ARID1A-deficient HCC cells markedly synthetically lethal. This renders their survival heavily dependent on mitochondrial respiration (41).

The mechanisms involved in cuproptosis are well established in preclinical studies, and the translational potential of targeting this form of cell death in human disease is enormous. The impairment of copper metabolism observed in a variety of cancers provides a strong rationale for targeting this pathway. Importantly, cuproptosis-related genes (CRGs) such as *FDX1* and *DLAT* hold promise as biomarkers with both diagnostic and therapeutic significance. These markers may help to differentiate between tumors that rely on distinct metabolic pathways, allowing the precise targeting of specific subgroups of HCC (e.g., ARID1A-mutated or low-glycolytic tumors).

### 3.2 Copper and ferroptosis

Ferroptosis is an iron-dependent form of regulated cell death characterized by the accumulation of phospholipid peroxides, which leads to plasma membrane rupture and cell death. This process is initiated by elevated levels of ROS and iron-dependent lipid peroxidation. Numerous well-characterized signaling pathways and cellular processes contribute to the regulation of ferroptosis (43, 44). Targeting ferroptosis has shown great potential in cancer treatment (45).

Despite evidence implicating iron accumulation as a key driver of ferroptosis (8), emerging evidence indicates that copper also plays a significant role in promoting this form of cell death, particularly in HCC. Copper may either directly induce ferroptosis or sensitize cells to it through a variety of mechanisms (46). For example, ceruloplasmin, a copper-binding protein, facilitates the oxidation of Fe²^+^ to Fe³^+^, promoting iron loading onto transferrin and its cellular uptake *via* transferrin receptors. This copper-driven enhancement of iron metabolism increases intracellular iron and mitochondrial oxidative stress, thereby promoting ferroptosis in different cancer cell types (46, 47). Cu^2+^ enhances the ubiquitination of glutathione peroxidase 4 (GPX4) as well as the formation of GPX4 aggregates by directly binding to the cysteine residues C107 and C148 in the GPX4 protein. The autophagy receptor tax1 binding protein 1 (TAX1BP1) facilitates the breakdown of GPX4 under copper-induced stress, leading to ferroptosis (48).

One study suggested that the ferroptosis-inducing agents sorafenib and erastin boost cuproptosis in primary liver cancer cells by promoting the aggregation of copper-dependent lipoylated proteins (49). Mechanistically, sorafenib and erastin increase protein lipoylation by inhibiting the mitochondrial matrix-related proteases responsible for the breakdown of FDX1, and lowering the production of the intracellular copper chelator glutathione (GSH) by inhibiting the intake of cystine (49). In addition to acting as a cofactor for GPX4, GSH also functions as a copper chelator, thereby inhibiting copper deposition. Accordingly, a decline in GSH concentrations favors enhanced copper accumulation in cells (46). Liu S. et al. reported that DLAT oligomerization and ROS accumulation following GSH depletion mediated by the nanocomplex CaCO3/Mn/Cu@lip-Apt promoted cuproptosis (50). This indicated the existence of a feedback loop between cuproptosis and ferroptosis.

**Figure 3:**
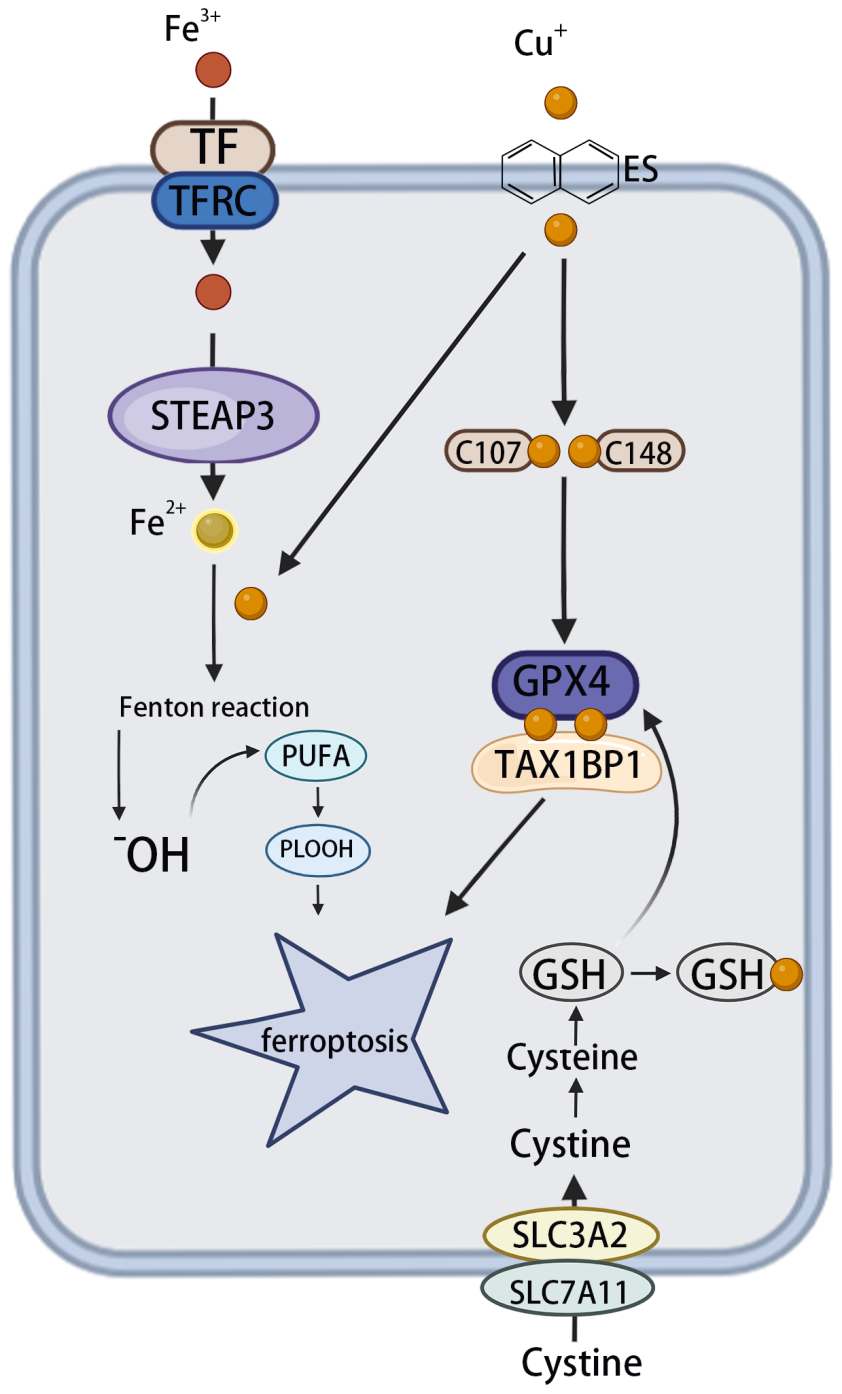
The effect of copper on ferroptosis. The circulating apoprotein TF binds to the TFRC and facilitates the endocytosis of Fe^3+^. This is followed by the conversion of Fe^3+^ to Fe^2+^ in endosomes, which is mediated by STEAP3. OH is produced *via* the Fenton reaction, which, in turn, leads to lipid peroxidation and the phenomenon known as “ferroptosis”. Meanwhile, copper can facilitate the Fenton reaction, leading to the formation of PLOOH and, consequently, ferroptosis. Cu^+^ directly binds to the C107 and C148 cysteine residues of the GPX4 protein, inducing its aggregation. These aggregates are subsequently recognized by the autophagy receptor TAX1BP1 and degraded *via* the autophagy pathway, with ferroptosis occurring in response to autophagy. TF, transferrin; TFRC, transferrin receptor; STEAP3, six transmembrane epithelial antigen 3; OH, hydroxyl radicals; PLOOH, phospholipid hydroperoxides; GPX4, glutathione peroxidase 4; TAX1BP1, tax1-binding protein 1.

These findings contribute to the understanding of the interrelationship between copper-induced and iron-induced cell death. However, most current studies describe only a single pathway and lack a comprehensive mechanistic map. In-depth investigations are needed to elucidate the molecular basis of the synergy or antagonism between these two processes (**Figure 3**).

### 3.3 A comparison between cuproptosis and other forms of programmed cell death

Copper-induced cell death was first reported in the 1980s (51). Before this, it was believed that copper ionophores used in cancer treatment, such as disulfiram (DSF) and eletroxin, elicited cell death through the induction of mitochondria-dependent apoptosis (52). It was suggested that the Cu^2+^-elesclomol complex was transported to mitochondria, where Cu^2+^ underwent reduction to Cu^+^, resulting in ROS-dependent cell apoptosis (53). Yadava et al. found that Cu^2+^-elesclomol effectively oxidizes physiological concentrations of ascorbic acid, generating harmful H_2_O_2_ through the reaction of Cu^+^ with O_2._ Subsequently, the reaction between H_2_O_2_ with Cu^+^ produces more destructive and highly reactive ROS (54). Overall, cuproptosis was initially classified as conventional oxidative stress-induced cell death (e.g., apoptosis or necrosis).

Studies have show that cell death induced by elesclomol does not involve the cleavage or activation of caspase-3, which is a hallmark of apoptosis (55). The ability of elesclomol to kill cells remained intact when BAX and BAK1, key effectors of apoptosis, were knocked out, or when cells were treated with pan-caspase inhibitors (Z-VAD-FMK and Boc-D-FMK) (7). This suggests that cuproptosis is distinct from apoptosis. Additionally, the use of other inhibitors of known cell death mechanisms, such as ferrostatin-1 and necrostatin-1, was ineffective in eliminating copper ionophore-induced cell death (7). This implied that the mechanism involved in cuproptosis differed from those of other known cell death types. Accordingly, in 2022, Tsvetkov et al. formally introduced the concept of “cuproptosis” (7). Although additional studies have since confirmed that cuproptosis is distinct from classical cell death pathways (e.g., FDX1-dependent DLAT aggregation), its independence and physiological relevance require further exploration. For instance, the molecular mechanisms underlying copper-induced DLAT aggregation have not been fully elucidated, and *in vivo* studies may involve cross-effects of secondary cell death pathways. Furthermore, the ability of *in vitro* models using supraphysiological concentrations of copper to mimic pathological copper homeostasis needs validation. These questions await confirmation through evidence of unique regulators and *in vivo* characterization.

Ferroptosis is largely dependent on GSH inactivation. However, in tumors, complex compensatory mechanisms are activated that maintain GSH levels (56, 57). This significantly reduces the therapeutic efficiency of GSH pathway-targeting strategies. HCC cells are metabolically characterized by aerobic respiration (OXPHOS) and glycolysis, with a preference for the latter, known as the Warburg effect (58). This metabolic profile suggests that proliferating HCC cells preferentially use glycolysis even in the presence of O_2_ (59). A hypoxic tumor microenvironment (TME) induces the accumulation of hypoxia-inducible factor 1 alpha (HIF-1α), which blocks the activation of the NLRP3 inflammasome and the necrosome (60, 61). This greatly limits the therapeutic efficacy of targeting certain forms of programmed cell death, such as apoptosis and necroptosis, in HCC. Notably, patient-derived HCC cells and clinical samples usually show different responses to programmed cell death, which may be related to differences in hypoxic gradients and genetic backgrounds (61, 62). Future studies should integrate complementary models to better assess the relevance of necroptosis and apoptosis in HCC treatment (**Table 1**). The heterogeneity of programmed cell death responses emphasizes the need for reliable biomarkers, such as GSDM family proteins for pyroptosis, RIPK3 for necroptosis, and p53/GPX4 for ferroptosis (69). However, the crosstalk between programmed cell death pathways *in vivo*—where a single biomarker, such as p53, may be involved in multiple processes—complicates their precise differentiation. This underscores the significance of identifying pathway-specific exclusion markers (e.g., oligomeric DLAT in copper protrusions) to elucidate the mechanisms of concurrent cell death in complex microenvironments.

**Table 1:** Comparative analysis of cell death mechanisms.

Feature	Key trigger factors	key controlling gene	Core molecular mechanism	Cancer treatment potential	Ref
Cuproptosis	Copper accumulation(Cu²^+^).	FDX1,DLAT,LIAS	Cu²^+^ binds to mitochondrial lipoylated proteins. Induction of DLAT oligomerization & iron-sulphur cluster protein degradation. Proteotoxic stress.	Targeting high mitochondrial respiratory tumors (high FDX1 expression).	(7, 36)
Ferroptosis	Iron ion accumulation.	GPX4,ACSL4,SLC7A11,p53	GPX4 inactivation → lipid ROS accumulation. ACSL4 mediates PUFA phospholipid synthesis.	Targeting GPX4 low expression tumors.	(63–65)
Pyroptosis	inflammatory vesicles activation.	NLRP3,Caspase-1	Caspase-1/4/5/11 cleaves GSDMD. GSDMD-N-terminal pore formation → cellular pyroptosis. IL-1β/IL-18 release.	Enhancement of anti-tumour immunity (release of inflammatory factors).	(66, 67)
Necroptosis	TNFα/RIPK1 signaling activation.	caspase-8,MLKL, RIPK3,RIPK1	RIPK1-RIPK3-MLKL phosphorylation. MLKL oligomerization → plasma membrane perforation	Overcoming apoptosis resistance.	(68)

In contrast, tumour cells exhibit heightened sensitivity to copper ions, a phenomenon attributable to the active mitochondrial respiratory chain and the frequent expression of genes related to copper-induced death (e.g., DLAT) in HCC cells. Himoto et al. identified a positive correlation between HIF-1α and copper levels in HCC patients, suggesting that activation of HIF-1α due to copper accumulation may promote hepatocarcinogenesis and tumour progression (70). This observation underscores the merits of copper-induced death as a treatment modality for HCC when compared with other forms of programmed cell death. It also sheds light on a potential therapeutic strategy for HCC involving the combined use of copper ion carriers and HIF-1α inhibitors to overcome hypoxia-mediated HCC progression and drug resistance (70).

## 4 Inhibitory effect of copper on HCC

### 4.1 The role of copper in the regulation of angiogenesis

The progression of tumors and tumour metastasis is inextricably linked to dysregulated angiogenesis (71, 72). Consequently, its inhibition within tumors has long been a key strategy in cancer treatment, particularly in the liver, where the blood supply is abundant. Hypoxia represents a crucial microenvironmental factor influencing the rate of tumour angiogenesis. The main aspect of how cells adjust to low oxygen levels is the transcription factor HIF-1α (73). Copper was previously believed to possess pro-angiogenic properties due to its capacity to upregulate the expression of HIF-1α (74). However, the mechanisms by which copper regulates tumour angiogenesis are increasingly being revealed with continued research. It is now known that copper can inhibit tumour angiogenesis by binding to other metal-complexing ligands, and most current evidence suggests that copper depletion within the TME represents an effective strategy for inhibiting tumour angiogenesis (75).

Vascular endothelial growth factor (VEGF) and its membrane-localized receptor (VEGFR) play a pivotal role in tumour-induced angiogenesis (72). Copper and its associated regulatory molecules were shown to contribute to the activation of several pro-angiogenic factors, including VEGF, tumour necrosis factor alpha (TNF-α), IL-1, IL-6, and IL-8 (9, 76). CTR1 plays a major role in the reparative angiogenesis induced by developmental cues, VEGF, ischemia, or wounding (77). CTR1 deficiency results in the blockade of VEGF signaling and angiogenesis in endothelial cells (ECs) (77). Yee et al. demonstrated that the knockdown of CTR1 in a related model resulted in a reduction in intracellular copper levels (78). Similarly, ATOX1 transports copper ions to the *VEGF* promoter, which is closely related to the regulation of angiogenesis (78). Studies have indicated that low levels of ATOX1 effectively reduce copper transport capacity within endothelial cells, resulting in a significant decrease in copper ion transport to sites contributing to cellular functions, such as the *VEGF* promoter, ultimately inhibiting angiogenesis (79). Although this process leads to the accumulation of intracellular copper ions, low levels of ATOX1 mean that copper ions do not reach relevant cellular functional sites (78). Consequently, reducing intratumoral copper levels and inhibiting the expression of relevant pro-angiogenic factors still offers a promising therapeutic strategy for cancer.

### 4.2 The modulatory role of copper in HCC metastasis

Metastasis is a complex process that occurs in several stages and depends on both intrinsic traits and extrinsic microenvironmental factors. Cancer cells exhibit higher proliferation and division rates than normal cells, resulting in a greater demand for copper.

CRGs, such as *CDKN2A* and *GLS*, play a crucial role in cancer spread. Transwell cell migration analysis suggested that the upregulation of the expression of both genes is associated with the capacity to promote HCC cell metastasis (80). In HCC cells, a positive correlation was found between MYC levels and the amount of intracellular copper. MYC regulates the transcription of *SLC31A1*, which is involved in copper homeostasis. Elevated copper levels, in turn, were found to promote tumour growth and metastasis in a mouse model (46, 81). In summary, CRGs exhibit clinical translational potential as biomarkers for early diagnosis and stratification of HCC metastasis risk through the regulation of copper homeostasis and metastatic cascade responses.

Copper activates metastasis-related enzymes and cascade reactions in cancer. Epithelial-mesenchymal transition (EMT) is an embryonic phenotypic plasticity program. In tumors, this process can be reactivated, which allows cancer cells to acquire characteristics such as aggressiveness, dissemination ability, and resistance to chemotherapy/immunotherapy (82). The non-classical TGF-β signaling pathway is among the pathways that can activate EMT. TGF-β plays a central role in tumour metastasis by stimulating PI3K and its downstream pathways through the phosphorylation of AKT and the promotion of the EMT signaling pathway. Conversely, copper depletion significantly inhibits EMT pathway activation (32). Jiang et al. reported that EMT can be promoted by upregulating TGF-β, which, in turn, drives HCC metastasis (83). This suggests that targeting the copper-TGF-β axis (e.g., copper carriers combined with TGF-β inhibitors) might reverse the EMT phenotype and inhibit HCC metastasis (84). In addition, copper depletion has been shown to downregulate EMT-related proteins associated with cancer invasion and metastasis in TNBC, NB, and DIPG cell lines, and to decrease metastasis *in vivo (* 32, 84).

Copper is a key component of several metalloenzymes, including matrix metalloproteinase 9 (MMP-9), SOD1, vascular adhesion protein-1 (VAP-1), and LOX, which are essential for cancer metastasis (85–88). SOD3 belongs to a class of secreted copper-containing enzymes with a dual role in tumour metastasis, that is, both its over- and underexpression promote the development of invasive mesenchymal carcinoma (22, 89). LOX plays a significant role in tumour metastasis through the production of hydrogen peroxide (H_2_O_2_), which activates Src/FAK signaling and the EMT process (90, 91). Ramchandani D. et al. showed that tetrathiomolybdate (TM) significantly inhibited lung and breast cancer metastasis by chelating copper ions, thereby reducing cellular invasion, *in vitro* metastasis, and the metastatic burden (92). Notably, copper chelator treatment alone was not sufficient to fully deplete copper in tumour cells, possibly due to compensatory mechanisms such as transporter protein plasticity (CTR1/ATP7A, etc.) and HIF-1α-mediated glycolytic metabolic reprogramming (78, 93, 94).

In summary, targeting copper metabolism can inhibit cancer metastasis. Combination therapies based on the copper chelator TM (e.g., synergistic LOX/TGF-β inhibitors) have the potential to reduce tumour resistance to copper depletion by effectively overcoming adaptive resistance. This approach holds potential for limiting HCC progression and metastasis, providing a new direction for clinical translation. However, the precise regulatory mechanism and therapeutic strategies involved require in-depth validation.

### 4.3 Copper and oxidative stress

Oxidative stress results from an imbalance between the production of highly reactive free radicals and oxidants and their removal by the antioxidant system. This imbalance damages important biological molecules (lipids, proteins, and genetic material) and cellular structures, which disrupts how the cell functions and its overall stability (6). Copper deficiency can impair the antioxidant system (95).

Copper dishomeostasis induces oxidative stress in both directions. Copper overload induces DLAT oligomerization by triggering cuproptosis, leading to a burst of ROS. Conversely, copper deficiency inhibits the hepatic Nrf2 signaling pathway (a classical antioxidant regulatory hub) and significantly reduces antioxidant enzyme activity, inducing oxidative stress and leading to liver injury (6, 95). Thus, copper has a dual role in the oxidative response. Elevated ROS levels are believed to have an oncogenic effect, leading to DNA damage and chromosomal instability, which, in turn, activates proto-oncogenes and deactivates tumor suppressor genes (96, 97). Accordingly, oxidative stress can also exert pro-apoptotic effects on HCC cells. For instance, Cu(sal)(phen) induces apoptosis in a dose-dependent manner in HCC cells by increasing ROS production in mitochondria (98). To reduce ROS overproduction in the tumour immune microenvironment, cancer cells engage antioxidant defense mechanisms. These include the activation of the KEAP1-Nrf2 pathway and the regulatory control of GSH metabolism, thereby supporting cell survival and proliferation (95, 99).

In summary, oxidative stress exhibits a double-edged effect in cancer, exerting both pro-tumorigenic and anti-tumorigenic effects (100). However, unregulated oxidative stress can damage normal tissues, highlighting the need for the precise targeting of cancer cells to trigger oxidative stress for tumour therapy. Reis et al. demonstrated the therapeutic potential of targeting ROS by selectively inhibiting NADPH oxidase (NOX)—a transmembrane enzyme involved in ROS production—to trigger tumour-specific oxidative stress (101). Further identification of tumour-specific oxidative stress biomarkers employing multi-omics and mechanism-oriented clinical trials will be essential for assessing the feasibility of such targeted therapies.

### 4.4 Copper and the tumor immune microenvironment

Tumor progression is closely associated with abnormalities in the immune system. The programmed death receptor 1 (PD-1)/programmed death ligand 1 (PD-L1) signaling pathway is recognized for its role in promoting cancer cell survival (102). Cancer cells can avoid being destroyed by T cells through PD-1 and PD-L1 immune checkpoints, which help them escape the immune system (102). This results in reduced T-cell survival, proliferation, and function in the TME, which weakens anti-cancer immunity (103). Copper-targeted modulation of the immune microenvironment potentiates PD-1/PD-L1 blockade therapy, and its homeostatic mechanism represents a new target for immunotherapy (104). Voli F. et al. demonstrated that elevated copper levels in cancer patients are positively correlated with PD-L1 upregulation, suggesting that copper accumulation promotes tumour immune escape (105).

Liao M et al. built a predictive signature model based on Cu-binding proteins, termed the CuPscore (106). They found that HCC patients with high CuPscores exhibited significantly lower proportions of monocytes, CD8+ T cells, γ+T cells, and mast cells, whereas those with low CuPscores demonstrated lower proportions of M0 macrophages. This suggested that both copper-binding proteins and copper play regulatory roles in the immune microenvironment and the immunotherapeutic response in HCC (106). Studies have demonstrated that copper depletion leads to the downregulation of STAT3, EGFR, AKT, and GSK3β phosphorylation, thereby inhibiting PD-L1 transcription and modulating PD-L1 ubiquitination and stabilization/degradation, ultimately promoting tumor suppression (105). However, copper levels exceeding a certain threshold have the same anti-tumour immune escape effect. Mechanistically, Cu^2+^ accumulation can trigger immunogenic cell death (ICD) in tumors by inducing cuproptosis in cancer cells, leading to the release of damage-associated molecular patterns (DAMPs). This, in turn, promotes anti-tumour immunity through the robust production of cytotoxic T cells (CD8) and T helper cells (CD4+) (107). Notably, the immunosuppressive hepatic microenvironment can counteract copper-regulated immunotherapeutic responses through certain mechanisms, such as the induction of CXCL12 and PD-L1 expression by the TGF-β1/Smad/SOX18 axis. This highlights the current therapeutic challenge presented by the copper-mediated tumour immune microenvironment in HCC (108).

Copper depletion (PD-L1 degradation) and copper accumulation (DAMP release) were shown to block tumour immune escape by different mechanisms, confirming the immunomodulatory potential of targeting copper. Additionally, Yuan et al. demonstrated that cuproptosis-related immune checkpoint genes (CRICGs) such as *IFNB1*, *CCR5*, and *STING* can predict the tumour immunosuppressive microenvironment. Their multi-omics analysis indicated that the expression profiles of these genes were significantly correlated with the response to immunotherapy (109). The continued identification of CRICGs is expected to provide novel biomarkers for predicting the sensitivity of HCC patients to immunotherapy.

## 5 Copper-mediated resistance to chemotherapeutic agents

The efficacy of sorafenib as a first-line drug for the treatment of HCC is often invalidated by the activation of molecules and pathways such as EGFR and AKT, leading to drug resistance (110). Banerjee K et al. found that copper chelators significantly inhibit the activation and expression of the EGFR/PI3K/AKT pathway, thereby overcoming multidrug resistance (111). These findings highlight the potential of targeting copper metabolism in overcoming sorafenib resistance in HCC. In addition, AMPK-mediated autophagy is also a cause of sorafenib resistance, with a meta-analysis showing that elevated copper levels lead to the activation of AMPK, suggesting that copper plays a regulatory role in sorafenib resistance (112). Notably, although prolonged AMPK activation enhances sorafenib resistance, the activation of AMPK nevertheless inhibits the invasive ability of cancer cells (92). Lu Y. et al. identified miR-3689a-3p as a key regulator of sorafenib sensitivity by CRISPR/Cas9 screening. They confirmed that miR-3689a-3p sensitized HCC cells to the effects of sorafenib by targeting the copper chaperone protein CCS, disrupting intracellular copper transport, and inhibiting SOD1-dependent mitigation of oxidative stress (113). This mechanism provides a novel basis for reversing drug resistance *via* copper targeting.

The role of long non-coding RNAs (lncRNAs) in HCC progression has been increasingly recognized over recent years. By targeting multiple miRNAs, lncRNAs can increase the levels of target mRNAs, thereby affecting HCC progression (114, 115). The deletion of LINC02362 in HCC cells has been demonstrated to enhance the survival, migratory ability, and invasiveness of HCC cells. Quan B et al. demonstrated that the FDX1 expression level was significantly correlated with cisplatin and oxaliplatin sensitivity, and that LINC02362 regulates its downstream gene *FDX1 via* hsa-miR-18a-5p (116). LINC02362 knockdown led to a reduction in copper concentrations and resistance to elesclomol-Cu (ES-Cu). This, in turn, suppressed FDX1 expression. Thus, the LINC02362/hsa-miR-18a-5p/FDX1 axis represents a novel pathway that drives cuprotosis, suppresses metastasis, and enhances sensitivity to oxaliplatin in HCC (116). While further exploration of the functions and potential mechanisms of action of lncRNAs in HCC is needed, it is nonetheless evident that they play a role in this disease.

The function of copper transporters in regulating the uptake, efflux, and distribution of cisplatin has been extensively documented in various cancers (117, 118). In several of them, including ovarian cancer, CTR1 expression levels are positively correlated with cisplatin sensitivity, with low expression increasing resistance, and high expression enhancing sensitivity (118, 119).

In addition, copper metabolism disorders and CRGs may modulate chemotherapy resistance through cross-pathways. For example, the secretion of IL-18 by pyroptotic macrophages activates the TLR4/NF-κB pathway, thereby inducing resistance to apoptosis, whereas the copper metabolism-related gene MURR1 domain 10 (*COMMD10*) antagonizes NF-κB activity by inhibiting TNF-α-mediated IκBα/p65 nuclear translocation (120, 121). This suggests that copper and genes related to copper-induced cell death can influence tumour immune resistance through crosstalk with other mechanisms.

In conclusion, the findings of the above-mentioned studies suggest that copper metabolism may bi-directionally mediate HCC chemoresistance by modulating the interactions between genes associated with copper-induced cell death, lncRNAs, and other cell death pathways. While mechanistic studies provide multidimensional validation, the paucity of clinical data hinders the translation of copper-targeting therapies. Copper-targeting intervention strategies (chelators such as TM, ionophores such as elesclomol) have shown significant potential for reversing drug resistance, and systematic preclinical trials are urgently needed to validate the safety and efficacy to drive precision clinical translation.

## 6 Functions and clinical implications of CRGs as biomarkers in HCC

Tumors are closely associated with cuproptosis. The multifaceted regulation of HCC by cuproptosis-associated genes highlights their notable potential in the diagnosis, treatment, and prognosis of this cancer.

FDX1 is a small iron-sulfur protein localized to the mitochondrial matrix with roles in steroidogenesis, Fe-S cluster biosynthesis, and lipoylation (122). Its expression is influenced by multiple factors, including microRNAs, transcription factors, and m^6^A mRNA modifications (116, 123, 124). During cuproptosis, FDX1 reduces divalent copper ions to monovalent ones, thereby enhancing cytotoxicity and cellular functions (125). It also acts as a central regulator of protein lipidation upstream of the lipoylation pathway (7). Quan Y et al. analyzed FDX1 expression data from The Cancer Genome Atlas (TCGA) for patients with HCC, and found that overall survival (OS), progression-free interval (PFI), disease-specific survival (DSS), and disease-free interval (DFI) were significantly better in patients with high FDX1 expression than in those with low FDX1 expression (126). Through a combination of bioinformatics analysis and *in vitro* and *in vivo* experiments, Sun B et al. demonstrated that FDX1 deficiency increases ROS levels and activates both mitochondrial autophagy and the PI3K/AKT signaling pathway in HCC cells (127). These studies clearly demonstrate the diagnostic and therapeutic potential of FDX1 in HCC.

DLAT is a vital subunit of the PDC (128), playing a pivotal role in the transfer of the acetyl group from pyruvate, formed during oxidative decarboxylation of pyruvate, to coenzyme A (CoA) (129). This results in the formation of acetyl-CoA, thereby serving as a crucial nexus that connects glycolysis to the TCA cycle. DLAT oligomerization leads to proteotoxic stress and results in cell death, which suggests that DLAT plays a significant role in cuproptosis (130). Employing data from TCGA, Yin Q et al. demonstrated that DLAT upregulation was associated with malignancy and poor prognosis in HCC, and that patients with high DLAT expression exhibited a tendency towards decreased OS (131). Wang N. et al. noted that the deletion of DLAT resulted in a reduction in leucine levels and, consequently, the inhibition of mTOR signaling. This, in turn, suppressed HCC proliferation and progression (132).

Pyruvate dehydrogenase B (PDHB) and pyruvate dehydrogenase E1 component subunit alpha 1 (PDHA1) constitute the β and α1subunits, respectively, of the PDC E1 component (133). PDHA1 plays a pivotal role in tumorigenesis by regulating the metabolic shift from OXPHOS to aerobic glycolysis (134, 135). PDHB drives the transcription of glycolysis-related genes and metabolic reprogramming by binding to the *SLC2A1*, *GPI*, and *PKM2* promoters, thereby inducing sorafenib resistance in HCC (136). Additionally, high expression of both PDHB and PDHA1 in HCC is suggestive of a poor prognosis (134, 136).

SEC14L3 is a member of the human SEC14-like (SEC14L) protein family within the phosphatidylinositol transfer protein (PITP) superfamily. Recent studies have shown that low expression of SEC14L3 in HCC is significantly associated with poor OS, DSS, and PFS in patients with HCC (137). Mechanistically, copper intracellular transport promoted by electrochlorohydrin upregulates SEC14L3 expression, which, in turn, enhances FDX1 expression *via* the ERK/YY1 axis, induces thiooctylation of DLAT, triggers its oligomerization, and ultimately leads to cell death and the inhibition of HCC growth (137).

The influence of cuproptosis on HCC, mediated by CRGs, suggests that copper-based therapies could provide more targeted and personalized treatment options for patients with HCC. This approach holds significant promise for the treatment of liver cancer (46) (**Table 2**).

**Table 2:** Overview of common CRGs.

Gene	Full name	Expression in HCC	Functions	Clinical values of HCC	Ref.
FDX1	Ferredoxin 1	Down	Ruduce Cu^2+^ to Cu^+^	High expression in HCC is associated with better prognosis	(126)
DLAT	Dihydrolipoamide S-acetyltransferase	Up	lipoylated DLAT oligomerization lead to cell death	High DLAT expression levels in HCC show a trend towards reduced OS	(131, 132)
PDHA1	Pyruvate Dehydrogenase E1 Subunit Alpha 1	Up	Inhibits the Warburg effect and induces apoptosis via a mitochondria-mediated pathway in HCC.	PDHA1 expression was significantly associated with survival prognosis only in HCC	(134, 135)
PDHB	Pyruvate dehydrogenase B	Up	Driving glycolytic gene transcription and metabolic reprogramming by binding to SLC2A1, GPI and PKM2 promoters, thereby inducing resistance to sorafenib in HCC.	High PDHB expression is significantly associated with high HCC tumour stage and grade and poor patient prognosis.	(136)
SEC14L3	SEC14-like protein 3	Down	Mediated copper deposition inhibits HCC growth *in vitro* and *in vivo* via ERK/YY1/FDX1 axis.	Low expression was significantly associated with OS, DSS and PFS.	(137)
CDKN2A	Cyclin Dependent Kinase Inhibitor 2A	Up	Enhances antioxidant defense and modulates lipoylated protein stability.	High expression is associated with poor prognosis and reduced immune infiltration in HCC.	(138)

## 7 Exploring cuprotosis-based therapeutic strategies for hepatocellular carcinoma

Imbalances in copper homeostasis are strongly associated with a variety of diseases. Copper chelators alleviate toxicity by binding and removing excess copper, while copper ion carriers facilitate copper uptake to induce cell death. Understanding the molecular mechanisms and therapeutic potential of the two classes of copper modulators may provide a theoretical basis for the development of copper metabolism-targeted drugs for the treatment of HCC (139) (**Table 3**).

**Table 3:** Comparison of copper chelators and copper ionophores.

Type	Drug	Effects OR Involved mechanisms	limitation	Ref
Copper chelators	Trientine	Binding of copper ions promotes biliary excretion and restores copper homeostasis; Inhibition of angiogenesis. Inhibit inflammatory response effectively during and after liver RFA(Radiofrequency ablation). Blocking copper absorption in the gastrointestinal tract.	Deterioration of neurological function. Gastrointestinal side effects.	(140–142)
	D-penicillamine (D-pen)	Chelation with Zn activates the cGAS-STING pathway to induce tumour cell death. D-pen binds to Cu generate ROS and inhibit ICAM and LOX.	Deterioration of neurological function. May interfere with essential metal homeostasis.	(143–145)
	Ammonium Tetrathiomolybdate (ATTM)	Inhibition of TRPV4/calcium/NF-κB signaling pathway attenuates hepatic oxidative stress and inflammatory injury. The combination of TM and HAL has great potential for the treatment of hepatocellular carcinoma by reducing tumour hypoxia and angiogenesis.	Copper deficiency due to long-term use. Need to monitor liver and kidney function.	(146–148)
Copper ionophores	Elesclomol (ES)	Transport of Cu²^+^ to mitochondria induces DLAT oligomerization and degradation of iron-sulfur cluster proteins.	Dependence on FDX1 expression and DLAT activity. Ineffective in HCC with defective mitochondrial function.	(125, 149)
	Disulfiram (DSF)	Forms complex CuET with Cu²^+^, which inhibits proteasome function and promotes ROS production. Depletes GSH to enhance oxidative stress. Induces ferroptosis. Inhibition of both NF-κB and TGF-β.	Binding to copper to form CuET causes systemic toxicity and side effects in non-tumour tissues. High-dose neurotoxicity.	(150–153)
	Salicylaldehyde isonicotinoyl hydrazone (SIH-1)	SIH-1 preferentially kill HepG2 cells superior to clioquinol. SIH-1/Cu^2+^ complex has stronger ability in releasing copper by GSH, inducing redox imbalance and triggering mitochondria-mediated apoptosis of HepG2 cells.	Lower stability constant.	(154)

### 7.1 Copper chelators

Copper chelators have been shown to suppress angiogenesis, metastasis, and tumour growth by binding to copper and reducing its bioavailability (48, 155). Several copper chelators have proven useful in lowering available copper, thus inhibiting blood vessel formation and, consequently, HCC development.

Trientine, a traditional selective copper chelator, has been demonstrated to suppress the role of copper as a pro-angiogenic cofactor, thereby exerting anti-angiogenic effects in HCC (140). One study demonstrated that trientine exerted a pronounced inhibitory effect on HCC neovascularization, resulting in a markedly lower number of CD31-immunopositive vessels compared to that observed in the control condition. A semiquantitative analysis showed that the number of CD-31-positive vessels was 4.2 times higher in the control group than in the trientine group (94). In addition, using trientine before treatment effectively reduced the inflammatory response during and after liver radiofrequency ablation (RFA) in live subjects (141). This suggests a novel approach for attenuating RFA-mediated liver inflammation and protecting liver tissue (141). Other copper chelators, including D-penicillamine (D-pen) and ammonium tetrathiomolybdate (ATTM), have also demonstrated therapeutic efficacy in both transplanted tumor models and clinical trials (143, 156–159) (**Table 3**).

However, copper chelators require combination therapies for optimal efficacy. Consequently, they must be administered in conjunction with other medications to enhance antitumor effects and thereby demonstrate their potential in cancer therapy (95).

### 7.2 Copper ionophores

In contrast to copper chelators, copper ionophores were shown to increase the bioavailability of intracellular copper, thereby triggering intracellular copper accumulation and, ultimately, also cuproptosis (**Table 3**).

#### 7.2.1 Disulfiram

DSF has been identified as a copper ion carrier with potential anticancer activity (46). DSF forms a complex with copper, which has demonstrable copper-dependent anticancer properties in both *in vitro* and *in vivo* models. Regarding mechanisms, DSF/Cu can inhibit the nuclear translocation of NF-κB subunits and the expression of Smad4, a key mediator of the NF-κB and TGF-β signaling pathways. This results in the downregulation of Snail and Slug, which inhibits EMT, and, consequently, HCC metastasis (160). Furthermore, DSF/Cu can target the NPL4 component of p97 separase, a ubiquitin-recognizing factor involved in the regulation and proteasomal turnover of various proteins in the stress response pathway, thereby promoting cancer cell death (46, 161). The antitumor properties of DSF are dependent on Cu binding, with the DTC-Cu complex (CuET) in DSF/Cu serving as the main component responsible for these effects (150). The copper-accumulating properties of HCC provide a “natural target” for DSF. In addition, DSF/Cu demonstrates a selective toxicity profile, exhibiting a higher degree of toxicity toward HCC cells than toward non-malignant ones (33). Conventional anticancer drugs, such as cisplatin and 5-fluorouracil (5-FU), have limited efficacy, and DSF has been shown to significantly enhance their antitumor effects (162). In a rat model of HCC, Hassan et al. administered 5-FU, along with copper or DSF. The combination of 5-FU and DSF led to a notable improvement in most parameters assessed, with 5-FU + Cu demonstrating a similar trend. This indicates that adjusting the Cu level significantly boosts the antineoplastic activity of 5-FU and makes cancer cells more responsive to the drug (6). ATTM and DSF are also pertinent in the context of cancer clinical trials. Both have been reported to facilitate the uptake of copper- and platinum-containing anticancer drugs, as platinum and copper share the same CTR1 copper transporter (163).

A retrospective epidemiological investigation was conducted on the risk of cancer-specific mortality for Danish patients diagnosed with cancer who received DSF between 2000 and 2013. The results indicated that patients who had previously undergone treatment with DSF for alcohol abstinence exhibited reduced mortality in comparison to those who had not received DSF (161). This finding supports the potential of DSF as a therapeutic drug for cancer. However, DSF also has some drawbacks. Research has demonstrated that the direct administration of DSF as a single agent (without Cu) did not produce the desired clinical benefit, possibly due to its rapid metabolism after ingestion (164). However, the addition of exogenous Cu^+^ may also pose risks of physiological toxicity. Moreover, the CuET produced by the DSF-Cu combination in non-tumour tissues can also cause systemic toxicity and side effects (151). Although DSF has been the subject of extensive study in clinical trials for a variety of cancers (**Table 4**), large-scale trials of its treatment for HCC are yet to be conducted, resulting in limited assessment of clinical efficacy.

**Table 4:** Clinical trials of Elesclomol or DSF/Cu in patients with cancer.

Drug use	Model	Study	Enrollment	Status	Dose	Study Completion	Clinical trial ID
DSF+Cu	Glioblastoma	PhaseII/III	88	Completion	DSF 400 mg p.o./d Cu 2 mg p.o./d	2021.01	NCT02678975
DSF	Germ-cell tumors	PhaseII	12	Completion	DSF 400mg p.o./d	2022.01	NCT03950830
DSF	Prostate Cancer	Not Applicable	19	Completion	Cohort 1: 250mg p.o./d Cohort 2: 500mg p.o./d	2012.06	NCT01118741
DSF+chelated zinc	Melanoma	PhaseII	12	Completion	N/A	2013.11	NCT02101008
DSF+Cu	Liver cancer	PhaseI	21	Completion	DSF 250 mg p.o./d Cu 2mg/4mg/6mg/8mg	2013.03	NCT00742911
DSF	Non-small Cell Lung Cancer	PhaseII/III	60	Completion	N/A	2009.12	NCT00312819
Elesclomol	Soft Tissue Sarcoma	PhaseII	80	Completion	N/A	2005.10	NCT00087997
Elesclomol+Paclitaxel	Persistent Ovarian Epithelial Cancer Fallopian Tube Cancer Primary Peritoneal Cancer	PhaseII	58	Completion	N/A	2016.08	NCT00888615
Elesclomol+Docetaxel	Prostate Cancer	PhaseI	34	Completion	N/A	2010.05	NCT00808418
Elesclomol+Paclitaxel	Melanoma	PhaseI/II	103	ongoing	N/A	N/A	NCT00084214
Elesclomol+Paclitaxel+Carboplatin	Stage IIIB Non-Small Cell Lung Cancer Stage IV Non-Small Cell Lung Cancer	PhaseI/II	86	Completion	N/A	2005.03	NCT00088088

#### 7.2.2 Elesclomol

Elesclomol is a copper ion carrier that has been extensively investigated for its anticancer effects. Elesclomol forms a strong 1:1 complex with Cu^2+^ and may exert its anticancer activity through the induction of oxidative stress and/or its ability to transport copper into cells (149). The efficacy of elesclomol in eradicating cancerous cells is contingent on its capacity to facilitate the extracellular transportation of copper ions, a pivotal step in cuproptosis (165). Moreover, the antitumor efficacy of this compound is also dependent on the extent to which cancer relies on mitochondria for energy production (165). Li et al. showed that the use of elesclomol resulted in a reduction in the expression of the mitochondrial outer membrane transport protein 20 (TOM20) and an increase in the formation of DLAT oligomers. These findings suggest that elesclomol may have a role in promoting cancer cell death (40).

Clinical trial data to date show no evidence of added toxicity for elesclomol, either in isolation or in combination with chemotherapy regimens (**Table 4**). The maximum tolerated dose was established as 438 mg/m² in a Phase I study conducted on patients with solid tumors. The adverse reaction profile that was observed at this dose level was found to be analogous to that of paclitaxel monotherapy (166). The low intrinsic toxicity of elesclomol in healthy cells suggests that the endogenous antioxidant capacity of such cells can overcome the potential toxicity of elesclomol (53). It is therefore encouraging to note that elesclomol has a good safety profile in cancer therapy, which supports further development of the drug.

However, clinical trials have shown that elesclomol has poor efficacy in oncological treatments, and there is a marked lack of clinical trials for HCC, which limits its therapeutic promise in this cancer (167). This limited efficacy may be partly attributable to its linear *in vivo* pharmacokinetics, characterized by rapid elimination from plasma (biological half-life: 1.06 ± 0.24 h) and low steady-state apparent volume of distribution (25.1 ± 8.1 L/m^2^) (168). Consequently, there is a growing emphasis on investigating strategies to augment the therapeutic efficacy of elesclomol. For example, based on the poor water solubility of elesclomol-Cu (ES-Cu), Wang Q et al. designed a d-α-tocopherol polyethylene glycol 1000 succinate/chondroitin sulfate-cholic acid (TPGS/CS-CA)-based nanomicelle to deliver ES-Cu complexes to a variety of cancer cells from different lines. The ES-Cu nanoparticles were packaged in these nanomicelles and were able to bypass P-gp without affecting its activity. P-gp is an efflux transporter protein that may contribute to multidrug resistance (MDR) in cancer cells (169). Wu et al. developed a ROS-responsive polymer (PCP) based on cinnamaldehyde and polyethylene glycol to encapsulate ES-Cu, forming ECPCP (170). ECPCP markedly prolonged the systemic circulation of ES-Cu and enhanced its tumor accumulation, thereby improving its dual antitumor mechanism (170).

## 8 Conclusions and future perspectives

Cuproptosis, a recently identified form of cell death distinct from necroptosis, ferroptosis, and pyroptosis, holds great promise for inhibiting tumour proliferation, reversing drug resistance, and facilitating early cancer diagnosis and treatment by modulating the lipid acylation modification of TCA cycle-associated proteins. The liver, a key organ in copper metabolism, is extremely sensitive to an imbalance in copper levels. This implies that cuproptosis has significant potential in the treatment of HCC, an effect that can be facilitated using a variety of strategies, including targeting copper accumulation for oncostatic effects or exacerbating the risk of toxicity by interfering with hepatic copper metabolism. However, this field still faces major challenges. The morphology of cuproptotic cells has not been systematically described, and the molecular mechanisms underlying this form of cell death (e.g., copper ion-specific activation mechanisms, downstream signaling networks) have not been adequately investigated, which severely limits its clinical translation.

The dysregulation of copper homeostasis can lead to serious toxic side effects, and there is an urgent need to develop precise strategies for monitoring and regulating copper levels to ensure therapeutic safety. Emerging nanocarrier delivery systems can significantly optimize the pharmacokinetic properties of copper-modulated drugs (e.g. copper chelators and ionic carriers), providing new ideas for achieving safe and effective copper homeostatic modulation through mechanisms such as prolonged half-life, enhanced targeting, and reduced systemic toxicity. However, rigorous validation through multicenter clinical trials is required to ensure the targeted delivery efficiency, assess the off-target effects, and evaluate the long-term biocompatibility of these systems. At the mechanistic level, although the functional roles of CRGs such as *FDX1*, *DLAT*, *CDKN2A*, and others have been predicted based on bioinformatics association analysis using databases such as TCGA and GEO, these genes lack sufficient functional and experimental support in molecular pathways. A more complex question relates to the possible interaction between copper-induced apoptosis and programmed cell death pathways such as ferroptosis. However, how these cell death pathways interact with each other to orchestrate the metabolic stress response requires further elucidation through spatiotemporal dynamic analyses.

With regard to model testing, the core bottlenecks in current research are the difficulty in simulating the physiological regulatory network of copper metabolism in the human liver in traditional animal models and the scarcity of liver-specific gene editing models, which limits the depth of mechanistic analysis. Emerging organoid technology offers a novel approach to overcoming this challenge by facilitating precise editing of copper metabolism core genes in liver organoids through the use of CRISPR-Cas9. In conjunction with microfluidic organoids, a perfusion-type microenvironment can be constructed, enabling dynamic simulation of the entire process of copper ion uptake, intracellular transport, and bile excretion. The integration of transcriptome-proteome-metabolome analysis allows the construction of high-fidelity disease models and the high-throughput screening of copper-targeted drugs and gene therapy vectors. Future research must focus on the standardization of patient-derived organoid banks, as well as analyzing the *trans*-tissue regulation of copper homeostasis through the hepatic-biliary-vascular multi-organ microarray system, and ultimately to promote the clinical translation of individualized anti-HCC therapy based on the mechanism of cuproptosis.

## Supplementary material

Supplementary Table 1 Search strategy.
