# Supplementary material for: Copper metabolism and cuproptosis: broad perspectives in the treatment of hepatocellular carcinoma
Source: Front Oncol. 2025 Jul 30;15:1555858. doi: 10.3389/fonc.2025.1555858 (PMC12343259; doi:10.3389/fonc.2025.1555858)
Supplement: Supplementary Table 1 — Search strategy. [file Table1.pdf]

Table S1. Search strategy

No.	Search items
#1	"Carcinoma, Hepatocellular"[Mesh]
#2	((((((((((((((((((Carcinoma, Hepatocellular[Title/Abstract]) OR (Carcinomas, Hepatocellular[Title/Abstract])) OR (Hepatocellular Carcinomas[Title/Abstract])) OR (Hepatocellular Carcinoma[Title/Abstract])) OR (Hepatoma[Title/Abstract])) OR (Hepatomas[Title/Abstract])) OR (Liver Cancer, Adult[Title/Abstract])) OR (Adult Liver Cancer[Title/Abstract])) OR (Adult Liver Cancers[Title/Abstract])) OR (Cancer, Adult Liver[Title/Abstract])) OR (Cancers, Adult Liver[Title/Abstract])) OR (Liver Cancers, Adult[Title/Abstract])) OR (Liver Cell Carcinoma[Title/Abstract])) OR (Carcinoma, Liver Cell[Title/Abstract])) OR (Carcinomas, Liver Cell[Title/Abstract])) OR (Cell Carcinoma, Liver[Title/Abstract])) OR (Cell Carcinomas, Liver[Title/Abstract])) OR (Liver Cell Carcinomas[Title/Abstract])) OR (Liver Cell Carcinoma, Adult[Title/Abstract])
#3	#1 OR #2
#4	"Copper"[Mesh]
#5	((Copper[Title/Abstract]) OR (Copper-63[Title/Abstract])) OR (Copper 63[Title/Abstract])
#6	cuproptosis[Title/Abstract]
#7	#4 OR #5 OR #6
#8	#3 AND #2
